# Characteristics of the Cerebrospinal Fluid in Septic Patients with Critical Illness Polyneuropathy - A Retrospective Cohort Study

**DOI:** 10.2478/jccm-2024-0018

**Published:** 2024-04-30

**Authors:** Yanyang Zhang, Jinfu Ma, Qing Zhao, Hui Liu

**Affiliations:** 1^st^ Medical Center of Chinese PLA General Hospital, Beijing, China; The 305^th^ Hospital of Chinese PLA, Beijing, China

**Keywords:** acute physiology and chronic health evaluation II, cerebrospinal fluid, critical illness polyneuropathy, sepsis, sequential organ failure assessment score

## Abstract

**Background:**

Critical illness polyneuropathy (CIP) is a complex disease commonly occurring in septic patients which indicates a worse prognosis. Herein, we investigated the characteristics of cerebrospinal fluid (CSF) in septic patients with CIP.

**Methods:**

This retrospective study was conducted between Match 1, 2018, and July 1, 2022. Patients with sepsis who underwent a CSF examination and nerve electrophysiology were included. The levels of protein, glucose, lipopolysaccharide, white blood cell (WBC), interleukin (IL)-1, IL-6, IL-8, and tumor necrosis factor (TNF) α in CSF were measured. The fungi and bacteria in CSF were also assessed.

**Results:**

Among the 175 septic patients, 116 (66.3%) patients were diagnosed with CIP. 28-day Mortality in CIP patients was higher than that in non-CIP patients (25.0% vs. 10.2%, P = 0.02) which was confirmed by survival analysis. The results of propensity score matching analysis (PSMA) indicated a significant difference in the level of protein, WBC, IL-1, IL-6, IL-8, and TNFα present in the CSF between CIP patients and non-CIP patients. The results of the receiver operating characteristic (ROC) analysis showed that IL-1, WBC, TNFα, and their combined indicator had a good diagnostic value with an AUC > 0.8.

**Conclusion:**

The increase in the levels of WBC, IL-1, and TNFα in CSF might be an indicator of CIP in septic patients.

## Introduction

Sepsis is a critical situation with high mortality in the intensive care unit. Critical illness polyneuropathy (CIP) is a complex disease that affects 30%–70% of critically ill patients [1], and the percentage of affected patients is 70%–80% when they suffer from sepsis or multiple organ dysfunction syndrome (MODS) [2]. Some studies found that septic patients with CIP had a longer length of stay (LOS), longer duration of positive pressure ventilation (PPV), higher medical expenses, and poorer prognosis [3, 4, 5]. The complications of CIP include akinesis, expectoration weakness, lung infection, deep vein thrombosis (DVT), and loss of lean body mass [6]. These complications are associated with the effect of systemic inflammation response syndrome (SIRS) in sepsis. However, studies on the influence of inflammation storm on the cerebrospinal fluid (CSF) of septic patients with CIP are limited. Therefore, the changes in CSF need to be elucidated to better understand its characteristics. In this study, we investigated the characteristics of CSF in septic patients with CIP.

## Methods

### Study population

**Fig. 1.**
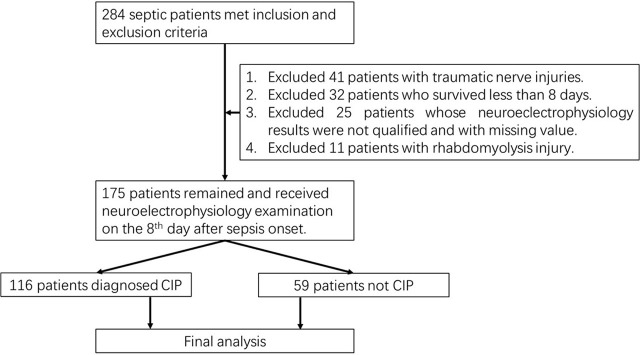
Workflow of enrollment. In total, 116 septic patients with CIP and 59 patients without CIP were included. CIP, critical illness polyneuropathy.

The institutional ethics committee of the General Hospital of the People's Liberation Army approved this single-center retrospective cohort study (S2018–718–02). In total, 175 septic patients were admitted to our Critical Care Medicine department from March 2018 to July 2022, including 94 male and 81 female patients. The process of patient enrollment is shown in Figure 1. The ages of the patients ranged from 18 to 80 years. The inclusion criteria were as follows: (1) a diagnosis of sepsis based on the latest guidelines (sepsis 3.0) **[7]**; (2) age ≥18 years and ≤80 years; and (3) sepsis caused by abdominal infection, intestinal puncture, obstruction, fistulation or traumatic injury, biliary system infection, pancreatitis infection, or intestinal flora dysfunction. The exclusion criteria were as follows: (1) patients with a history of nervous system diseases, such as Guillain-Barre syndrome, multiple sclerosis, epilepsy, and cerebral infarction; (2) toxic injury, which can impair nerve conduction, including lead, mercury, arsenic, thallium, alcohol, barbital poisoning, bacterial toxin, and animal toxin, such as diphtherin, tetanus, and tetrodotoxin; (3) malnutrition, especially vitamin B deficiency; (4) usage of muscle relaxant; (5) deep sedation; (6) severe limb injury preventing electrophysiology examinations; (7) survival time less than eight days without electrophysiology examination; (8) intracranial infection, such as bacterial meningitis and other intracranial infections; and (9) demyelinating diseases or neuromuscular junction dysfunction.

### Demographic and clinical data

The following demographic and clinical data were collected from each patient on the day of sepsis onset: age; gender; diagnosis; Acute Physiology and Chronic Health Evaluation (APACHE II) score; Sequential Organ Failure Assessment (SOFA) score; blood lactate; blood sugar; albumin; white blood cell (WBC); body mass index (BMI); hemoglobin (Hb), and C responsive protein (CRP). The information on ICU length of stay (LOS), hospital LOS, and mechanical ventilation (MV) were collected from electronic medical records. For each patient, electromyography data were collected on the eighth day of the diagnosis of sepsis. CSF was collected and examined two to four days after sepsis onset. The levels of protein, WBC, interleukin (IL)-1, IL-6, IL-8, and tumor necrosis factor (TNF) α in CSF were measured.

### CIP diagnosis and electromyography measurement

All examinations were performed by a physician expert in electromyography and an assistant using KEY-POINT 7033A (Total functional electromyographic potentiometer, Denmark Dandy Co., Skovlunde,, Denmark). The electromyography examination was carried out on the 8^th^ day of sepsis onset. CIP was diagnosed according to the definition provided by Bolton (2005) **[8]**. The CIP diagnosis criteria were as follows: (1) in 2 or more nerves, the wave amplitudes of compound muscle action potential (CMAP) and sensory nerve action potential (SNAP) were at least 80% lower than the normal lower limit in electromyography; and (2) excluding critical illness myopathy (CIM), confirmed by an increase in creatine kinase (CK) and needle electromyography (EMG) showing myogenic abnormality, or other possible diseases except CIP. Median nerves, ulnar nerves, and common peroneal nerves underwent motor and sensory neurophysiological examination. Tibial nerves underwent motor neurophysiological examination. Common peroneal nerves underwent sensory neurophysiological examination. The motor neurophysiological examination included conduction velocity and CMAPamplitude, whereas the sensory neurophysiological examination included conduction velocity and SNAP amplitude.

### Statistical analysis

The R statistical software (version 4.1.2) was used to analyze the data. Normally distributed data were expressed as the mean ±standard deviation (±SD); the differences between groups of normally distributed data were determined using Student's *t* test. Dichotomous data were expressed as a number (percentage); the differences between groups of dichotomous data were determined using the Chi-square test. The sample size was calculated based on the data of CSF. The values of α and β were set as 0.05 and 0.1, respectively. The power of the test was set as 0.9. A bilateral difference test was conducted. The sample size was not more than 36 for each group after calculation. 59 non-CIP patients and 116 CIP patients were enrolled herein. The sample size of the study met the standard. A multivariate logistical regression model and receiver operating characteristic (ROC) curve were used to analyze the factors associated with CIP. Propensity score matching analysis (PSMA) **[9]** was conducted to adjust the covariates of SOFA, APACHE II, diabetes history, and lactate. The matched ratio was 1:1 and the method was ‘nearest’. Kaplan-Meier survival curve analysis was used to compare the survival rate within 28 days between the CIP group and the non-CIP group. All results were considered statistically significant at *P* < 0.05 (two-tailed).

## Results

### Clinical data

In total, 175 septic patients were included, of which 116 patients (66.3%) were diagnosed as CIP. The differences in age, gender, history of diabetes, cause of sepsis, hemoglobin, WBC, blood sugar, and albumin were not significant between CIP patients and non-CIP patients. Significant differences were recorded in lactate levels (*P* < 0.01), the SOFA score (*P* < 0.01), and the APACHE II score (*P* < 0.01). We also found that the prognosis of CIP patients was worse than that of non-CIP patients. The CIP patients had a longer ICU LOS (*P* = 0.01), a higher ratio of MV assistance (*P* = 0.01), and a higher mortality in 28 days (*P* = 0.02) (Table 1).

**Table 1.** Demographics and Clinical Data

**Characteristics**	**CIP Patients**	**Non-CIP Patients**	**P**
Counts	116	59	

Cause of sepsis			0.77
Intestinal puncture or fistulation	21 (18.1)	10 (16.9)	
Colon obstruction or fistulation	23 (19.8)	9 (15.3)	
Pulmonary infection	16 (13.8)	9 (15.3)	
Severe pancreatitis	9 (7.8)	3 (5.1)	
Thoracic and mediastinum infection	7 (6.0)	2 (3.4)	
Bile duct infection	3 (2.6)	3 (5.1)	
Intrauterine infection	3 (2.6)	2 (3.4)	
Diarrhea associated with antibiotics	7 (6.0)	6 (10.2)	
Liver abscess	7 (6.0)	8 (13.6)	
Spleen rupture and infection	6 (5.2)	2 (3.4)	
Blood stream infection	14 (12.1)	5 (8.5)	

General information			
Age (years)	57.7±15.0	56.9±15.4	0.76
Female	49 (42.2)	32 (54.2)	0.13
Diabetes history	39 (33.6)	28 (47.5)	0.08
BMI (kg/m^2^)	21.7±4.2	20.8±3.7	0.17
Hemoglobin (g/L)	110.2±20.1	112.1±17.5	0.54
WBC (× 10^9^/L)	10.8±2.9	10.5±3.1	0.54
Blood sugar (mmol/L)	8.1±3.3	7.8±3.7	0.59
Albumin (g/L)	27.4±7.9	28.3±7.3	0.47
Lactate (mmol/L)	7.5±4.2	5.2±3.4	<0.01
SOFA	11.5±4.6	9.2±4.0	<0.01
APACHE II	19.0±5.9	16.4±5.1	<0.01

Outcomes			
MV assistance	67	22	0.01
Death in 28 days	29	6	0.02
ICU length of stay (day)	19.4±8.6	16.5±5.3	0.01
Hospital length of stay (day)	27.2±16.1	23.9±7.7	0.08

Data are n (%) and mean ± standard deviation (±SD). APACHE II, acute physiology and chronic health evaluation II; BMI, body mass index; CIP, critical illness polyneuropathy; ICU, intensive care unit; MV, mechanical ventilation; SOFA, sequential organ failure assessment; WBC, white blood cell.

### Electromyography of the patients

Nerve injury was symmetric in CIP patients. Left limbs were selected to perform electromyography. We examined the median nerves, ulnar nerves, tibial nerves, peroneal nerves, and sural nerves in all 175 selected patients. In the upper limb, 163 nerves were diagnosed as abnormal, while in the lower limb, 171 nerves were diagnosed as abnormal (Table 2).

**Table 2.** Data of Electromyography

**Variables**	**Amplitude Decrease**		**Conduction Velocity Decrease**		**Abnormal Nerves**
	**Motor**	**Sensory**	**Motor**	**Sensory**	
Upper limb (n, %)					163 (93.1)
Median	57 (32.6)	106 (60.6)	27 (15.4)	15 (8.6)	
Ulnar	55 (31.4)	52 (29.7)	26 (14.9)	50 (28.6)	

Lower limb (n, %)					171 (97.7)
Tibial	72 (41.1)	-	49 (28.0)	-	
Common peroneal	95 (54.3)	-	58 (33.1)	-	
Sural	NA	31 (17.7)	-	11 (6.3)	

Data are n (%).

### CSF analysis in septic participants by PSMA

Significant differences were recorded in the lactate level (*P* < 0.01), SOFA score (*P* < 0.01), and APACHE II score (*P* < 0.01) between CIP patients and non-CIP patients (Table 3). PSMA was performed to adjust the influence of lactate, SOFA, and APACHE II. Patients were matched with a 1:1 ratio. Based on PSMA, 59 patients were selected in the CIP group and 59 patients were selected in the non-CIP group. The distribution of propensity scores was shown in Supplementary File 1. Significant differences in lactate, SOFA, and APACHE II were removed by PSMA (*P* > 0.05). Significant differences in CSF protein, WBC, IL-1, IL-6, IL-8, and TNFα were also recorded between CIP patients and non-CIP patients (Table 3).

**Table 3.** CSF Measurement in Septic Participants Before and After PSMA

**Characteristics**	**Before PSM**				**After PSM**			

	**All (n=175)**	**CIP (n=116)**	**Non-CIP (n=59)**	**P**	**All (n = 118)**	**CIP (n=59)**	**Non-CIP (n=59)**	**P**
Age (year)	57.4±15.1	57.7±15.0	56.9±15.4	0.76	57.3±15.3	57.7±15.4	56.9±15.4	0.79
SOFA	10.8±4.5	11.5±4.6	9.2±4.0	<0.01	9.5±4.1	9.7±4.3	9.2±4.0	0.52
APACHE II	18.1±5.8	19.0±5.9	16.4±5.1	<0.01	16.5±5.0	16.7±4.9	16.4±5.1	0.77
Lactate (mmol/L)	6.7±4.1	7.5±4.2	5.2±3.4	<0.01	5.5±3.5	5.8±3.7	5.2±3.4	0.41
Diabete history	67	39	28	0.11	56	29	27	0.85

CSF								
Glucose (mmol/L)	3.98±1.11	3.98±1.12	3.96±1.10	0.99	4.0±1.2	4.1±1.2	4.0±1.1	0.69
Protein (mg/L)	1369.3±841.0	1705.8±744.2	707.6±593.2	<0.01	1123.5±772.1	1539.4±706.2	707.6±593.2	<0.01
Cl-(mmol/L)	123.4±4.9	123.0±5.0	124.1±4.7	0.16	123.9±5.0	123.7±5.3	124.1±4.7	0.62
WBC (× 10^6^/L)	3.2±1.6	4.0±1.5	1.5±0.8	<0.01	2.8±1.6	4.0±1.2	1.5±0.8	<0.01
Lipopolysaccharide (eu/mL)	0	0	0	NA	0	0	0	NA
fungus	0	0	0	NA	0	0	0	NA
bacteria	0	0	0	NA	0	0	0	NA
Interleukin-1 (pg/m L)	23.0±12.6	29.4±10.0	10.5±5.8	<0.01	20.0±12.3	29.4±9.5	10.5±5.8	<0.01
Interleukin-6 (pg/m L)	1089.9±526.4	1264.1±429.0	747.3±535.1	<0.01	993.9±534.7	1240.4±408.5	747.3±535.1	<0.01
Interleukin-8 (pg/mL)	9558.9±4715.4	10949.5±3818.1	6824.7±5131.1	<0.01	8471.6±4838.6	10118.5±3921.8	6824.7±5131.1	<0.01
Tumor neucrosis factor-α (pg/mL)	17.7±7.2	21.7±4.1	9.9±5.2	<0.01	15.6±7.4	21.3±4.2	9.9±5.2	<0.01

Data are mean ± standard deviation (±SD). APACHE, acute physiology and chronic health evaluation; CSF, cerebral spine fluid; Cl−, chloride; PSM, propensity score matching; PSMA, propensity score matching analysis; SOFA, sequential organ failure assessment; WBC, white blood cell.

### Survival analysis of CIP and non-CIP patients

Within 28 days after the onset of sepsis, 29 patients (25.0%) died in the CIP group and six patients (10.2%) died in the non-CIP group; the 28-day mortality in the CIP group was higher than that in the non-CIP group (*P* = 0.02) (Table 1). The percentage of patients receiving mechanical ventilation was higher in the CIP group than in the non-CIP group. Patients in the CIP group had longer ICU LOS (*P* = 0.01). The difference in hospital LOS between the CIP group and the non-CIP group was not statistically significant (*P* = 0.08).

**Fig. 2.**
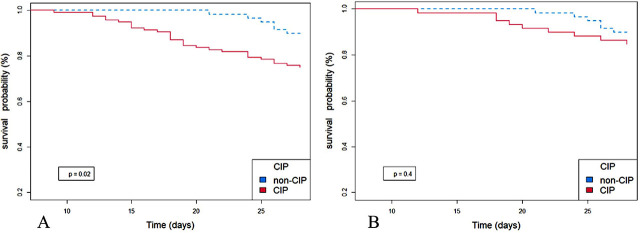
Survival analysis was conducted before and after PSMA. A. Survival analysis before PSMA; the 28-day mortality was higher in the CIP group than in the non-CIP group (P = 0.02). B. Survival analysis after PSMA; no significant difference was found between the CIP and non-CIP groups (P = 0.4). CIP, critical illness polyneuropathy; PSMA, propensity score matching analysis.

To show the difference in survival, the Kaplan-Meier survival curves were plotted for the 28-day survival rate of CIP patients and non-CIP patients (Figure 2). Because the patients who lived less than 8 days were excluded, the observation window was from day 8 to day 28. Our results showed a significant difference in the 28-day mortality between groups (*P* = 0.02) before PSMA, but not after PSMA (*P* = 0.4).

### Logistic regression analysis of the risk factors for CIP

Conditional logistical regression was used to investigate the best predictor for septic CIP. The results showed that the WBC (*P* = 0.006), IL-1 (*P* = 0.009, and TNF-a (*P* = 0.002) levels in CSF were significantly related to septic CIP. However, no differences in lactate (*P* = 0.26), SOFA (0.36), and age (*P* = 0.78) were found (Table 4).

**Table 4.** Conditional Logistical Regression Analysis

**Variables**	**β**	**OR**	**95% CI**	**Z value**	**Wald chi-square values**	**P**
Lactate	0.083	1.087	0.941–1.256	1.13	1.27	0.26
SOFA	0.059	1.061	0.934–1.204	0.91	0.83	0.36
Age	0.004	1.004	0.979–1.03	0.29	0.08	0.78

CSF						
Protein	0.001	1.001	0.998–1.004	0.46	0.21	0.64
WBC	2.688	14.696	2.204–98	2.78	7.71	0.006^*^
IL-1	0.337	1.401	1.089–1.801	2.62	6.88	0.009^*^
TNF-a	0.426	1.531	1.165–2.012	3.056	9.34	0.002^*^

* P<0.05. CI, confidence interval; CSF, cerebral spine fluid; IL, interleukin; OR, odds ratio; TNF, tumor necrosis factor; WBC, white blood cell.

### Prognostic value of CSF indicators for CIP

**Fig. 3.**
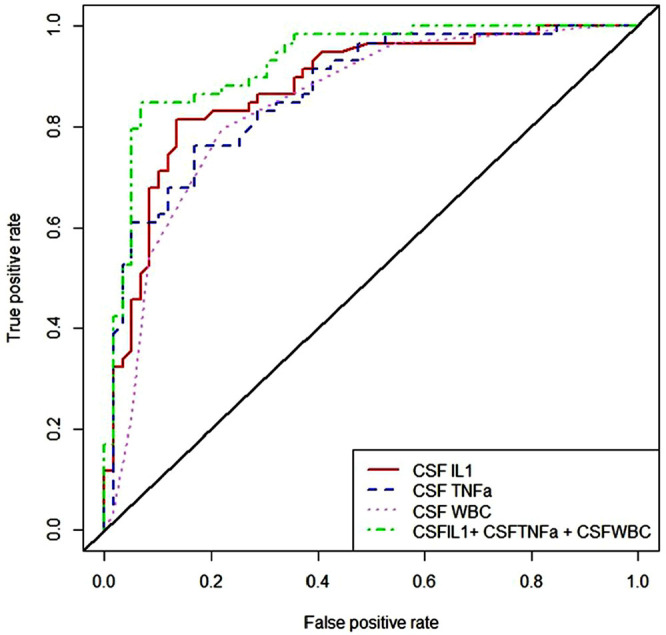
ROC analysis of three indicators and their combined mode used in the diagnosis of CIP. The AUCs of IL1, TNF-α, WBC, and IL1+ TNFa + WBC, were 0.878, 0.867, 0.843, and 0.926. ROC, receiver operating characteristic; AUC, area under curve; CIP, critical illness polyneuropathy; CSF, cerebrospinal fluid; IL, interleukin; TNF, tumor necrosis factor; WBC, white blood cell.

The results of the logistical regression analysis showed that the level of WBC, IL-1, and TNFα in CSF was significantly related to the occurrence of CIP (Table 4). Thus, the correlation between these factors and the prognosis of CIP was analyzed by receiver operator characteristic curve (ROC) analysis. The results showed that these indicators could effectively predict CIP (Figure 3 and Table 5). The combined indicator of WBC, IL-1, and TNFα had the highest AUC of 0.926. Details on the sensitivity, specificity, and cut-off value are provided in Table 5.

**Table 5.** ROC Analysis of 3 Indicators and Their Combined Mode in CSF

**Variables**	**Cut-off value**	**AUC (95% CI)**	**P**	**Sensitivity (%)**	**Specificity (%)**
IL1 (pg/mL)	19.250	0.878 (0.815–0.941)	<0.01	81.4	86.4
TNF-α (pg/mL)	17.050	0.867 (0.803–0.931)	<0.01	76.3	83.1
WBC (× 106/L)	2.500	0.843 (0.772–0.914)	<0.01	79.7	78.0
IL1+ TNFa + WBC	41.900	0.926 (0.879–0.974)	<0.01	84.3	93.2

AUC, area under curve; CSF, cerebrospinal fluid; IL-1, interleukin 1; ROC, receiver operating characteristic curve; TNF, tumor necrosis factor; WBC, white blood cell.

## Discussion

Neurologic disturbances are a common problem in septic patients in intensive care units, which are often associated with inflammatory injury induced by the systemic inflammation response syndrome in sepsis. The infiltration of immune cells and proinflammatory mediators into the nervous system plays a key role in nerve damage [10]. Based on this pathophysiological rationale, the central nervous system (CNS) should also be affected simultaneously by the inflammation storm in sepsis, as same as the peripheral nervous system (PNS). We found a novel relationship between septic CIP and changes in CSF, which provided deeper insights into the underlying pathogenesis of CIP and might help develop new diagnostic methods.

In this study, 175 septic patients were included. All participants underwent electromyography examination and CSF detection. In total, 163 nerves of the upper limb and 171 nerves of the lower limb were found to have abnormalities via electromyography. Also, 116 patients (66.3%) were diagnosed with critical illness polyneuropathy (CIP). For all participants, the SOFA score was 10.8 ±4.5, and the APACHE II score was 18.1 ±5.8; these values were similar to those reported in other studies. Some studies have shown that CIP occurs in approximately 30%–50% of ICU patients, with a higher incidence of 67% among critically ill patients with sepsis [11]. Our results showed that CIP patients had a higher lactate level, SOFA score, and APACHE II score, longer ICU stay, and higher 28-day mortality than non-CIP patients [12]. Diabetes is also a risk factor for nerve injury. The ratio of patients with a history of diabetes in both groups was similar (P = 0.08). Some researchers have found that the main reason that CIP patients have a poorer prognosis may be related to a prolonged mechanical ventilation duration, lower lung defense, high risk of deep vein thrombosis, and dysbiosis [1]. To increase the comparability between the groups, PSMA was used to remove the difference in the lactate level, SOFA score, and APACHE II score. The results showed that significant differences were present in the level of protein, WBC, interleukins (1, 6, and 8), and TNF-α in CSF between the CIP and non-CIP groups. Further analysis revealed that IL-1, TNF-α, and WBC had an AUC over 0.8 for predicting the occurrence of CIP. Our findings suggested that inflammation in CSF was correlated with CIP in patients with sepsis. This indicated that the inflammation storm also changed the composition of CSF, and CSF inflammation indicated peripheral nerve injury. This was the first study to report this finding.

The pathogenesis of CIP is not clear, and it may be closely related to the inflammatory response of the body, internal environment disturbance, cytokines, mitochondrial dysfunction, nerve damage due to reactive oxygen species, and hyperglycemia [13, 14]. Among them, the prominent causes include sepsis, systemic inflammatory response syndrome, and inflammatory factor damage [15]. The results of this study showed that CSF levels of multiple inflammatory factors, including IL-1, 6, 8, TNF-α, and WBC were significantly higher in CIP patients than in non-CIP patients, in the original cohorts and PSMA cohorts. The neurotoxic effects of these proinflammatory mediators have been reported previously. Inflammatory cytokines increase vascular permeability and enhance the penetration of neurotoxic inflammatory factors through the blood-nerve barrier, which leads to the development of CIP [16]. Similar changes occur in the brain. An inflammation cascade is triggered as proinflammatory mediators invade intracerebrally through a damaged blood-brain barrier. Thus, CSF is influenced simultaneously by peripheral nerves. This is why inflammation in CSF is also a strong signal of CIP, but the underlying mechanism remains unclear. However, treatments for CIP with glucocorticoids and immunoglobulin are either ineffective [17, 18] or controversial [19, 20]. These CIP patients had difficulty in getting rid of mechanical ventilation and were associated with prolonged stay at the ICU, higher medical costs, and increased long-term complication rates [12]. Our results confirmed these findings. In this study, CIP patients had higher 28-day mortality, longer ICU stays, and a higher percentage of treatment with mechanical ventilation.

The standard methods for the diagnosis of CIP are nerve electrophysiological examination and tissue biopsy. These operations cannot be performed easily in clinical practice. Some researchers proposed that CIP is a multiple lesion of peripheral nerve axons [21]. Histological examinations confirmed axonal degeneration of non-distal sensorimotor fibers, which can further decrease neuromuscular stimulation and result in muscle atrophy [22, 23, 24]. Schmidt et al. found that the Medical Research Council (MRC) score can be used to identify patients with CIP during intensive care bedside assessment [5]. As the levels of proinflammatory factors in CSF increased, we hypothesized that these factors might be used to differentiate between CIP patients and non-CIP patients. Based on our results, we speculated that evaluating these factors from the CSF of patients might help in diagnosing CIP in patients with sepsis. However, further investigation is required to confirm these findings.

### Limitations

First, this was a single-center, retrospective study; thus, patient selection bias might exist. However, we had rigid inclusion and exclusion criteria for enrolling participants. These steps helped achieve adequate consistency and decreased the heterogeneity of the study. Additionally, PSMA was used to increase the comparability between the CIP and non-CIP groups, which efficiently decreased the bias of a retrospective cohort study. Second, the sample size was relatively small, which probably affected the strength of the statistical analysis. This was probably why we obtained a negative result and found no difference in the 28-day mortality between the patients in the CIP and non-CIP groups in the survival analysis after PSMA. The sample size of this study met the standard, as determined by statistical calculations. Third, data on pathological biopsy were not used in this study. Nerve biopsy is the gold standard for the diagnosis of CIP. Thus, some non-CIP patients in this study may have been misdiagnosed as CIP. Nerve biopsy is an invasive operation and is difficult to perform. Thus, more time is required to collect the data on enough participants for statistical analysis. To resolve this problem, we recommend a multicenter, prospective study in the future.

## Conclusion

There was significant difference in CSF levels of multiple inflammatory factors (IL-1, TNF-α, and WBC) between CIP and non-CIP groups. Increment of inflammatory factors in CSF was a potential signal of the occurrence of CIP. This indicated inflammation storm in sepsis impacts both peripheral and central nervous system which helped shed more light on the pathogenesis of CIP.

## References

[1] Intiso D, Centra AM, Bartolo M, Gatta MT, Gravina M, Di Rienzo F (2022). Recovery and long term functional outcome in people with critical illness polyneuropathy and myopathy: a scoping review. BMC Neurol.

[2] Fan E, Cheek F, Chlan L (2014). An official American Thoracic Society Clinical Practice guideline: the diagnosis of intensive care unit-acquired weakness in adults. Am J Respir Crit Care Med.

[3] Latronico N, Rasulo FA, Eikermann M, Piva S (2023). Illness Weakness, Polyneuropathy and Myopathy: Diagnosis, treatment, and long-term outcomes. Crit Care.

[4] Cheung K, Rathbone A, Melanson M, Trier J, Ritsma BR, Allen MD (2021). Pathophysiology and management of critical illness polyneuropathy and myopathy. J Appl Physiol (1985).

[5] Schmidt D, Coelho AC, Vieira FN, Torres VF, Savi A, Vieira SRR (2019). Critical illness polyneuromyopathy in septic patients: Is it possible to diagnose it in a bedside clinical examination?. Arq Neuropsiquiatr.

[6] Tankisi H, de Carvalho M, Z'Graggen WJ (2020). Critical illness neuropathy. J Clin Neurophysiol.

[7] Singer M, Deutschman CS, Seymour CW (2016). The Third International Consensus Definitions for Sepsis and Septic Shock (Sepsis-3). JAMA.

[8] Bolton CF (2005). Neuromuscular manifestations of critical illness. Muscle Nerve.

[9] Benedetto U, Head SJ, Angelini GD, Blackstone EH (2018). Statistical primer: propensity score matching and its alternatives. Eur J Cardiothorac Surg.

[10] Wang Y, Guo L, Yin X (2022). Pathogenic TNF-α drives peripheral nerve inflammation in an Aire-deficient model of autoimmunity. Proc Natl Acad Sci U S A.

[11] Schorl M, Valerius-Kukula SJ, Kemmer TP (2013). Critical-illness-polyneuropathy as sequelae of severe neurological illness: incidence and impact on ventilator therapy and rehabilitation. NeuroRehabilitation.

[12] Kelmenson DA, Held N, Allen RR (2017). Outcomes of ICU patients with a discharge diagnosis of critical illness polyneuromyopathy: a propensity-matched analysis. Crit Care Med.

[13] Younger DS (2023). Critical illness-associated weakness and related motor disorders. Handb Clin Neurol.

[14] Guillouet M, Gueret G, Rannou F (2011). Tumor necrosis factor-alpha downregulates sodium current in skeletal muscle by protein kinase C activation: involvement in critical illness polyneuromyopathy. Am J Physiol Cell Physiol.

[15] Latronico N, Bolton CF (2011). Critical illness polyneuropathy and myopathy: a major cause of muscle weakness and paralysis. Lancet Neurol.

[16] Fisse AL, May C, Motte J (2021). New approaches to critical illness polyneuromyopathy: high-resolution neuromuscular ultrasound characteristics and cytokine profiling. Neurocrit Care.

[17] Apostolakis E, Papakonstantinou NA, Baikoussis NG, Papadopoulos G (2015). Intensive care unit-related generalized neuromuscular weakness due to critical illness polyneuropathy/myopathy in critically ill patients. J Anesth.

[18] Ydemann M, Eddelien HS, Lauritsen AØ (2012). Treatment of critical illness polyneuropathy and/or myopathy - a systematic review. Dan Med J.

[19] Brunner R, Rinner W, Haberler C (2013). Early treatment with IgM-enriched intravenous immunoglobulin does not mitigate critical illness polyneuropathy and/or myopathy in patients with multiple organ failure and SIRS/sepsis: a prospective, randomized, placebo-controlled, double-blinded trial. Crit Care.

[20] Elkalawy H, Sekhar P, Abosena W (2023). Early detection and assessment of intensive care unit-acquired weakness: a comprehensive review. Acute Crit Care.

[21] Diniz LRL, Portella VG, da Silva Alves KS (2018). Electrophysiologic alterations in the excitability of the sciatic and vagus nerves during early stages of sepsis. J Pain Res.

[22] Li Y. (2017). Axonal Sensorimotor Polyneuropathies. Continuum (Minneap Minn).

[23] Jung C, Choi NJ, Kim WJ (2020). Simplified diagnosis of critical illness polyneuropathy in patients with prolonged mechanical ventilation: a prospective observational cohort study. J Clin Med.

[24] Balke M, Teschler M, Schäfer H, Pape P, Mooren FC, Schmitz B (2022). Therapeutic potential of electromyostimulation (EMS) in critically ill patients-a systematic review. Front Physiol.

